# Clinical and microbiological outcomes of subgingival instrumentation supplemented with high-dose omega-3 polyunsaturated fatty acids in periodontal treatment – a randomized clinical trial

**DOI:** 10.1186/s12903-023-03018-7

**Published:** 2023-05-13

**Authors:** Mirella Stańdo-Retecka, Paweł Piatek, Magdalena Namiecinska, Radosław Bonikowski, Przemyslaw Lewkowicz, Natalia Lewkowicz

**Affiliations:** 1 Department of Periodontology and Oral Diseases, Medical University of Lodz, Ul. Pomorska 251, 92-213 Lodz, Poland; 2 Department of Immunogenetics, Medical University of Lodz, Ul. Pomorska 251/A4, 92-213 Lodz, Poland; 3 Faculty of Biotechnology and Food Sciences, Institute of Natural Products and Cosmetics, Lodz University of Technology, Ul. Stefanowskiego 2/22, 90-537 Lodz, Poland

**Keywords:** Periodontitis, Subgingival instrumentation, Non-surgical treatment, Eicosapentaenoic acid, Docosahexaenoic acid, Fish oil, Periodontal bacteria

## Abstract

**Purpose:**

This study aimed to evaluate the impact of dietary supplementation with omega-3 polyunsaturated fatty acids (PUFAs) eicosapentaenoic acid (EPA) and docosahexaenoic acid (DHA) combined with scaling and root planing (SRP) in untreated periodontitis stage III and IV.

**Methods:**

Forty patients were randomly assigned to the test group receiving SRP plus omega-3 PUFAs (*n* = 20) or control group receiving SRP alone (*n* = 20). Clinical changes of pocket probing depths (PD), clinical attachment level (CAL), bleeding on probing (BOP) and rates of closed pockets (PPD ≤ 4 mm without BOP) were evaluated at baseline and after 3 and 6 months. *Phorphyromonas gingivalis, Tanarella forsythia, Treponema denticola and Aggregatibacter actinomycetemcomitans* counts were analysed at baseline and at 6 months. Serum was subjected to lipid gas chromatography/mass spectrometry analysis at baseline and at 6 months.

**Results:**

Significant improvement of all clinical parameters at 3 and 6 months was observed in both groups. For the primary outcome “change of mean PD,” no significant difference was detected between the groups. Patients treated with omega-3 PUFAs demonstrated significantly lower rates of BOP, higher gain of CAL and higher number of closed pockets at 3 months in comparison to the control group. After 6 months, no clinical differences between the groups were found, with the exception of lower BOP rates. Moreover, in the test group, the number of key periodontal bacteria was significantly lower than in the control group at 6 months. Increased proportions of serum n-3 PUFAs and decreased proportions of n-6 PUFAs were detected at 6 months in the patients from the test group.

**Conclusion:**

High-dose omega-3 PUFA intake during non-surgical treatment of periodontitis results in short-term clinical and microbiological benefits.

The study protocol was approved by the ethical committee of Medical University of Lodz (reference number RNN/251/17/KE) and registered at clinicaltrials.gov (NCT04477395) on 20/07/2020.

## Introduction

Periodontitis is a chronic multifactorial disease induced by a microbial biofilm that is characterized by progressive destruction of the tooth-supporting apparatus [1]. The overall prevalence of the disease is estimated at approximately 45–50% [2]. Periodontal health is secured by homeostasis between a host and oral microbiome, while disturbances in homeostatic balance resulting from an aberrant host immune response or other inherited and/or acquired factors promote periodontitis [3]. Subgingival biofilm dysbiosis and overgrowth of gram-negative anaerobic species of bacteria lead to potent activation of immune response and, in a consequence, to periodontal tissue destruction and clinical manifestation of the disease [4].

The current strategy of treatment of periodontitis is based on the reduction of the subgingival biofilm that helps to reestablish homeostasis and health. This concept is based on the mechanical subgingival instrumentation, conventionally called scaling and root planing (SRP), combined with proper control of risk factors and home control of bacterial biofilm [5]. However, in some cases, subgingival debridement can be insufficient to compensate for the ongoing inflammatory process. Therefore, the wider interest is currently focused on the concept of including modulation of host response (host modulatory therapy, HMT) strategy into a treatment plan. The aim of HMT is to reduce a by-stander inflammatory damage to periodontal tissue, which in the long term, will help with tooth preservation and maintenance of oral esthetics and function [6]. A range of different drugs have been evaluated as host modulation agents, including nonsteroidal anti-inflammatory drugs, tetracyclines and bisphosphonates [7, 8]. However, these substances possess potential limitations and adverse effects when prescribed for HMT [7, 9, 10].

Diet is an important factor affecting periodontal health. A cross-sectional analysis in U.S. population showed that consumption of more pro-inflammatory diet was associated with moderate/severe periodontitis especially among older and male participants [11]. Likewise, Jauhiainen et al. showed that a poor-quality diet based on red meat, high intake of fat, salt, sucrose and alcohol was associated with periodontal pocket formation during the 11-year follow-up period [12]. Another study revealed a significant reduction in gingival bleeding in the experimental group consuming an anti‐inflammatory diet rich in omega‐3 fatty acids, vitamin C, vitamin D, antioxidants, plant nitrates and fibers, even in the absence of interdental hygiene [13]. Moreover, dietary intervention with fruits and vegetables, b-carotene, vitamin C, a-tocopherol, EPA and DHA in non-smoking patients with chronic generalized periodontitis, resulted in lower percentage of sites with PD > 3 mm after SRP [14]. Recently, European Federation of Periodontology (EFP) released clinical practice guideline for the treatment of stage I-III periodontitis [15]. Authors concluded that at present, it is unclear whether dietary counselling may improve periodontal treatment outcomes and additional research is needed. More recent study have also suggested that nutritional factors were correlated with staging and grading of periodontitis [16]. Specifically, consumption of yogurt, tofu, dark green vegetables, cabbage, napa cabbage showed negative weak correlations with stage, grade, PD, rate of PD 4–5 mm, BOP rate, PISA (periodontal inflamed surface area) and PISA/PESA (PESA, periodontal epithelial surface area).

A special interest in omega-3 PUFAs comes from their anti-inflammatory properties. Omega-3 PUFAs are natural substances that may be derived from oily fish, flaxseed oil, canola oil and walnuts. High-fat fish like mackerel or salmon can provide about 1.5 g – 3.0 g of omega-3 PUFAs per meal, whereas low-fat fish like cod can provide about 0.2 g – 0.3 g of omega-3 PUFAs per meal. Since Western diet is mainly rich in low-fat fish, omega-3 PUFA daily intake in Great Britain, Northern and Eastern Europe, Northern America or Australia is very low (0.1 g – 0.2 g) [17]. Therefore, dietary intake of EPA and DHA should be supplemented by fish oils (FO) or other sources.

Numerous studies demonstrated positive correlation between consumption of omega-3 PUFAs and reduction of inflammation in various inflammatory diseases, including periodontitis [18–23]. In periodontal patients, omega-3 PUFAs were administered at low doses in adjunction to mechanical periodontal debridement, in some studies with addition of low-dose acetylsalicylic acid [24–29]. The mechanism of action of omega-3 PUFAs relies predominantly on the inhibition of production of pro-inflammatory cytokines and eicosanoids. Next, omega-3 PUFAs are enzymatically transformed into anti-inflammatory lipid mediators, i.e. resolvins (RV) and protectins that can reduce by-stander tissue damage during inflammation [30]. Furthermore, consumption of omega-3 PUFAs offers additional benefits for general health. EPA and DHA derivatives possess anti-arrhythmic, anti-thrombotic and hypolipidemic effects, thus they provide cardiovascular protective effects and reduce the risk of death in the course of cardiovascular diseases [31–33].

Aside from the potential benefits of omega-3 PUFAs in the patients with periodontitis, the results of the clinical studies assessing their use in combination with non-surgical treatment are still inconclusive. Therefore, in the present randomized clinical trial we analyzed whether adjunctive administration of fish oil containing high-dose omega-3 PUFAs during non-surgical treatment of patients with untreated generalized periodontitis stage III and IV provides better clinical and microbiological outcomes than SRP alone.

## Materials and methods

### Study design and patient selection

The study was designed as a parallel-arm, randomized clinical trial. A six-month trial period was set: (i) baseline, (ii) 3 weeks (oral hygiene professional control); (iii) 3 months, (iiii) 6 months. The study protocol was approved by the Ethical Committee of Medical University of Lodz (reference number RNN/251/17/KE) and registered at clinicaltrials.gov (NCT04477395) on 20/07/2020. The study was conducted in accordance with the Declaration of Helsinki and all patients provided their informed consent. The study was performed between October 2017 and April 2021. Study participants were consecutively screened and enrolled at the Department of Periodontology and Oral Diseases, Medical University of Lodz by M.S. and N.L. Patients with severe generalized periodontitis fulfilling the criteria for stage III or IV periodontitis were invited to participate in the study. The participants were randomly assigned to one of the two treatment groups. The allocation sequence was generated by the investigator (P.L.) who was not involved in the recruitment of participants for the study by using a website www.randomization.com. Block randomization was used with the block size of four.

Following first step of periodontal therapy that included oral hygiene instructions and professional mechanical plaque removal [15], all patients were treated using non-surgical periodontal approach. Ultrasonic (Piezon Master 700, EMS) and hand (Mini Five Gracey Curette, Hu-Friedy) instrumentation were performed under a local anesthesia. During second step of therapy, two to four SRP sessions were performed dependent on the extend and advancement of periodontal disease. Debridement was continued until the root surface was clean and smooth, but no extensive root planing was performed. All participants were motivated and instructed how to maintain optimal oral hygiene at home. At week 3 after completion of initial SRP sessions, oral hygiene was re-examined and individual hygiene instructions were reinforced. Subgingival instrumentation was repeated at 3 months only at the bleeding sites with PD ≥ 4 mm as the third step of therapy.

Patients from the control group were treated by SRP only. Patients from the test group received additionally fish oil containing omega-3 PUFAs for 6 months. Subject allocation was concealed in sequentially numbered opaque sealed envelopes, and at 3 weeks after completion of baseline SRP sessions, envelopes were opened to reveal subject allocation to the clinician (M.S.) responsible for treatment. Fish oil (BioMarine Medical, Marinex International, Poland), derived from *Centroscymnus crepitater, Etmopterus granulosus, Deania colceai, Centrophorus scalpratus, Sardinops sagax, Scomber scombrus,* and *Gadus morhua* species, was administered twice a day at a dose of 10 ml starting three weeks after the completion of initial SRP sessions. Daily dose of 20 ml provided 2.6 g of EPA, 1.8 g of DHA, 1.4 g of alkylglycerols, 1.4 g of squalene, 240 µg of vitamin A and 2 µg of vitamin D3. Patients were asked to document daily fish oil intake and possible complaints in a diet diary. For better adherence, the participants had to reappear at the Department of Periodontology and Oral Diseases every 4 weeks to give back empty bottles and receive required bottles of the fish oil. Patients from both test and control groups were instructed not to change their usual dietary habits during the period of the trial.

Periodontal examination was performed by a periodontist (N.L.) blinded to the study allocation of patients. Treatments were performed by a PhD student (M.S.). Both periodontal examination and treatment were done under magnification using 2.7 × or 3.1 × loups.

### Eligibility and exclusion criteria

The inclusion criteria for subjects included presence of at least 18 scorable teeth (not including third molars), ≥ 4 teeth with PD ≥ 6 mm and CAL ≥ 5 mm, radiographic bone loss extending one-third of the root length, no periodontal treatment performed in the past. The exclusion criteria were as following: smoking, history of diabetes or chronic inflammatory disease, any diseases that compromise wound healing, history of radio- or chemotherapy, history of nonsteroidal anti-inflammatory drug (NSAIDs) intake > 3 days or use of antibiotics or corticosteroids 3 months prior to the study, a diet rich in omega-3 PUFAs or supplementation with omega-3 PUFAs.

### Clinical assessment

Clinical measurements were performed at three time points: at baseline, at 3 months and at 6 months. The following clinical parameters were assessed: full mouth plaque index (FMPI), bleeding on probing (BOP), probing depth (PD) and gingival recession (REC). Clinical attachment loss (CAL) was calculated as a sum of PD and REC at respective sites. All measurements were carried out using a manual 1-mm graduated periodontal probe (PCP-UNC 15, Hu-Friedy) and recorded at six sites. A trained and calibrated examiner (N.L.) performed all assessments at baseline and at all follow-up time points. The examiner reliability was high with agreement in assessment on all clinical parameters of above 80% (ICC > 0.80).

### Subgingival biofilm sampling

Subgingival biofilm collection was completed before any periodontal intervention following the removal of supragingival plaque and calculus using periodontal curettes. Samples for microbiological analysis were obtained by inserting a sterile number 30 paper endodontic point into the deepest periodontal pocket from each quadrant for 10 s. Samples were taken twice, at baseline before periodontal treatment and after 6 months. Samples were stored at -80 °C in sterile Eppendorf tubes until processing.

### Blood collection

At baseline and after 6 months, samples of blood were taken in sterile 3.5 mL tubes containing clotting activator. After 30 min when blood clotted, samples were centrifuged at 2000 × g for 15 min. at 4 °C degrees. Subsequently, serum was collected at 1.5 mL Eppendorf tubes and frozen at -80 °C.

### Quantification of Genomic DNA Expression with Droplet Digital PCR (ddPCR)

Paper points were placed into 300 µL sterile PBS and incubated overnight at 4° C with gentle shaking. Bacterial DNA was extracted using High Pure PCR Template Preparation Kit (Roche-Applied Science, Bavaria, Germany) following the manufacturer’s instructions. The genomic DNA quantification was performed with digital quantitative PCR using specific primers: *Porphyromonas gingivalis* (L16492) GATTGAAATGTAGATGACTGATGGTG_For and CCACACCTTCCTCACGCCT_Rev; *Aggregatibacter actinomycetemcomitans* (M75036) CCCTGGTAGTCCACGCTGTA_For and CACAAACCCATCTCTGAGTTCTTC_Rev; *Tannerella forsythia* (L16495) ACAGGGGAATAAAATGAGATACG_For and TTCACCGCTACACCACGC_Rev; *Treponema denticola* (JF700256) CATAAAGGTAAATGAGGAAAGGAGC_For and CATTCCCTCTTCTTCTTATTCTTCATC_Rev (Genomed, Warsaw, Poland) and QX200 droplet digital PCR system (Bio-Rad, Hercules, CA) with QX200™ ddPCR™ EvaGreen Supermix according to the manufacturer’s instructions. Digital PCR reaction was performed using 2 ng of total genomic DNA isolated from each sample. The results of digital quantitative PCR were analyzed with QuantSoft Analysis Pro v.1.0.596 (Bio-Rad) and expressed as the number of gene copies per sample.

### Serum lipid gas chromatography/mass spectrometry analysis

The analyzes were carried out in accordance with the previously described methodology [34]. Briefly, to the 50 mg of lyophilized samples, 200 µL of methyl tert-butyl ether (Merck KGaA, Darmstadt, Germany) and 200 µL of a 0.25-M solution of trimethylsulfonium hydroxide in methanol (Merck KGaA, Darmstadt, Germany) were added. Next, samples were incubated at 80 °C for 30 min, and gas chromatography/mass spectrometry was performed by GC/MS Pegasus 4D (LECO Corp. St. Joseph, Michigan, USA). The components were directly injected into the port of a GC/MS. The GC column Rt-2560 (length 100 m, internal diameter 0.25 mm, film thickness 0.20 µm; from Restek Corp., Bellefonte, USA, cat. No. 40602) was used. Then, 1 µL of the sample was applied to the split/splitless (SSL) injector in splitless mode (injector temperature 240 °C). The GC oven temperature was initially held at 140 °C for 5 min, and then increased to 240 °C for 30 min at a rate of 4 °C/min. Helium as a carrier gas was used at a flow of 1 mL/min. Mass spectra were collected using a time of flight mass spectrometer. The settings of mass spectrometry were as following: ion source temperature 200 °C, ionization energy 70 eV, and scan range 33 to 550 amu (atomic mass unit). Obtained mass spectra were compared with NIST/EPA/NIH and Wiley Registry of Mass Spectral Data mass spectral libraries.

### Statistical analysis

Sample size was calculated based on the primary outcome parameter (mean PD) from a study utilizing 300 mg of n-3 PUFAs during non-surgical periodontal treatment [25]. The study was powered at 80% to detect a mean PD difference of 0.6 mm (SD ± 0.53) between test and control group at 3 and 6 months. The minimum required sample size was calculated to be 14 patients for each group; to account for possible dropouts, 25 patients were enrolled for each group.

The statistical unit was the patient, and all sites with PD ≥ 4 mm at baseline were considered for the statistical analyses. The primary outcome variables were mean PD and PD change (Δ) at 3 and 6 months. Secondary variables were mean CAL, REC, BOP, FMPI and their changes (Δ), number of sites with PD ≥ 4 mm and BOP, the percent of closed pockets (PD ≤ 4 mm without BOP) at 3 and 6 months in relation to baseline, and number of key periopathogens and serum lipid concentrations at 6 months in relation to baseline.

All continuous variables were expressed as the mean ± SD. Statistical comparison of differences within the groups at three time points were determined by the one-way ANOVA test. Scheffe’s test was used for multiple comparisons as a post hoc test when statistical significances were identified in the ANOVA test. The differences between test and control groups were analyzed with the t-test. The Kolmogorov–Smirnov test and the Fisher’s test were used for verification of normal distribution and analysis of variances. The differences in the changes (Δ) from baseline to 3/6 months between the groups were analyzed using Mann–Whitney U test. All tests were conducted at the significance level of α ≤ 0.05.

## Results

### Supplementation with fish oil containing omega-3 PUFAs results in the short-term clinical benefits, especially in the deeper pockets (≥ 6 mm)

**Fig. 1:**
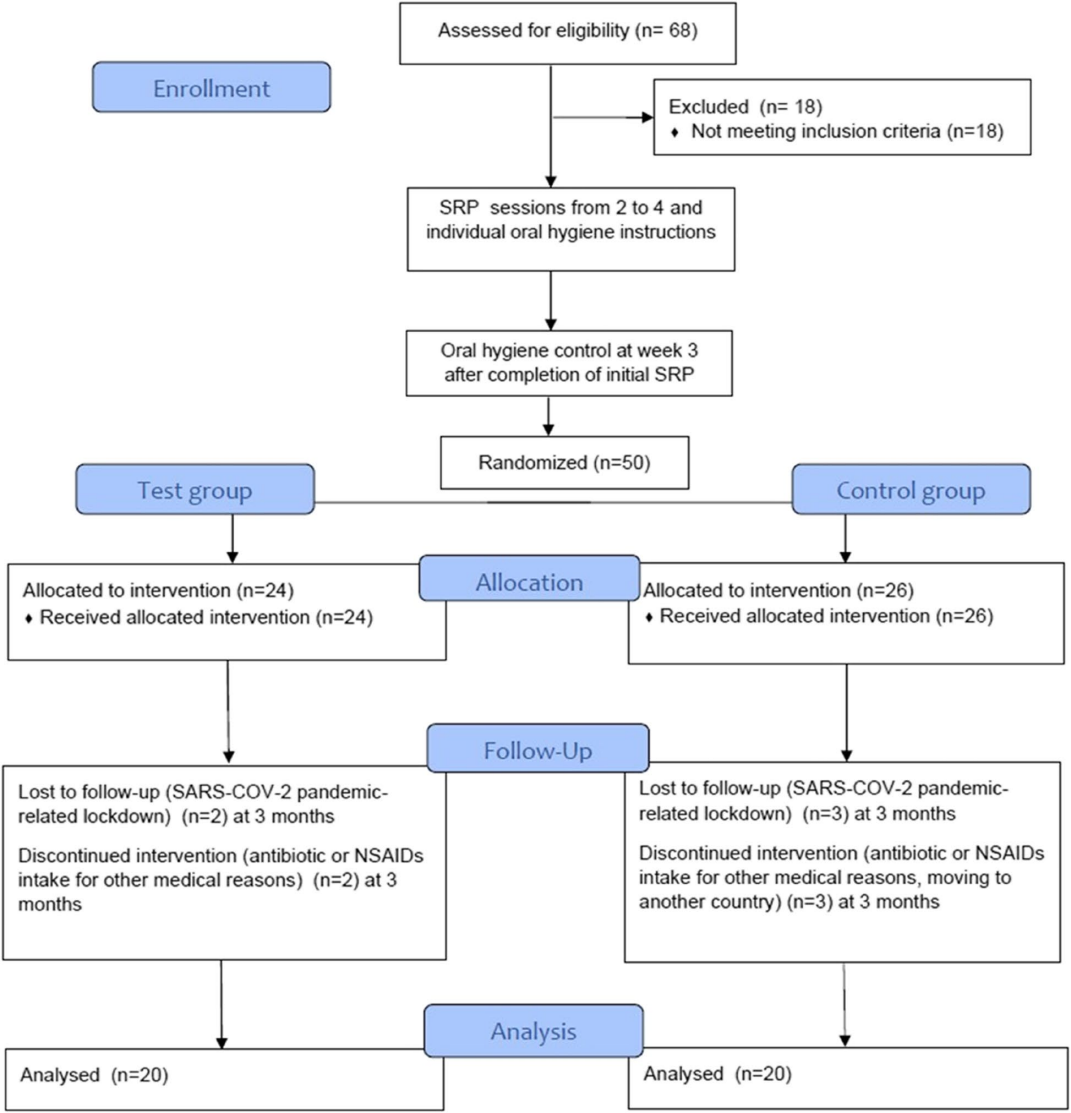
Study design/flow. SRP: scaling and root planing, NSAIDs: nonsteroidal anti-inflammatory drug, SARS-COV-2: severe acute respiratory syndrome coronavirus 2

A total of 68 individuals were screened and 50 patients (age range: 22–70 years; 25 females, 25 males; mean age 47.8 ± 10.7) with generalized periodontitis were finally included in the study. The test group consisted of 24 patients (12 males, 12 females, mean age 46.7 ± 9.4) and the control group consisted of 26 patients (13 males, 13 females, mean age 48.9 ± 11.9). Ten individuals (3 males/1 female from the test group and 2 males/4 females in the control group) were lost during follow-up because of the following reasons: antibiotic or NSAID intake for other medical reasons (*n* = 4), severe acute respiratory syndrome coronavirus 2 (SARS-COV-2) pandemic-related lockdown (*n* = 5), moving to another country (*n* = 1). No patients were lost due to the treatment protocol. In the intervention group, eight subjects reported nausea and irritating fish-scented halitosis that were not strong enough to stop the treatment regimen. No other adverse events were reported. Due to dropout, forty patients aged 30–70 years (mean age 48.2 ± 10.0 years) were available for evaluation after 6 months (Fig. 1).

All participants were diagnosed with generalized periodontitis stage III or IV according to the classification of periodontal diseases and conditions from 2017 [35]. Control group patients were older compared to the test group patients (52.2 ± 10.8 vs. 44.1 ± 7.7 years old) (Table 1).

**Table 1:** Demographic characteristics of the groups (without dropouts)

	**Test group *n* = 20**	**Control group *n* = 20**	***p* value**
Age (years; mean ± SD)	44.1 ± 7.7	52.2 ± 10.8	0.005
Sex (% of females)	55.0	45.0	n.s
Stage III grade B (%)	25	40	n.s
Stage III grade C (%)	35	30	n.s
Stage IV grade B (%)	20	20	n.s
Stage IV grade C (%)	20	10	n.s

*Abbreviations*: *n.s* non-significant

At baseline, periodontal measurements were comparable in both groups (Table 2). At 3 and 6 month, all clinical parameters significantly improved in both groups in comparison to the baseline. When compared between the groups for the primary outcome at 3 months, in the test group PD reduced from 5.01 ± 0.49 mm to 3.68 ± 0.68 mm, while in the controls from 5.02 ± 0.71 mm to 3.98 ± 0.69 mm, but the difference was not statistically significant (*p* = 0.12). Δ baseline–3 months PD difference was in favor of intervention group (1.33 ± 0.66 mm vs. 1.04 ± 0.48 mm), but not statistically significant (*p* = 0.06). At 6 months, additional improvement of PD was demonstrated in both groups compared to the baseline and 3 months, but the mean PD values were not significantly different (*p* = 0.28) between the test and control groups (3.49 ± 0.80 mm vs. 3.64 ± 0.82 mm). Similarly, the mean change (Δ) of PD in the test group compared to the control group was not significantly different at 6 months.

**Table 2:** Mean scores of clinical parameters and their Δ at baseline and 3 and 6 months in the test (SRP plus FO) and control (SRP alone) groups. Data are mean ± SD

Variables	Test group *n* = 20	Control group *n* = 20	Inter-group comparisons *p* value
No PD ≥ 4 mm			
Baseline	55.0 ± 24.6	50.0 ± 17.0	0.23
3 months	31.1 ± 19.8*	30.1 ± 16.2*	0.44
Δ baseline–3 months	23.9 ± 17.0	19.9 ± 8.01	0.17
6 months	27.2 ± 20.4*#	26.0 ± 17.0*#	0.42
Δ baseline–6 months	27.8 ± 18.2#	24.1 ± 12.7#	0.23
PD (mm)			
Baseline	5.01 ± 0.49	5.02 ± 0.71	0.48
3 months	3.68 ± 0.68*	3.98 ± 0.69*	0.12
Δ baseline–3 months	1.33 ± 0.66	1.04 ± 0.48	0.06
6 months	3.49 ± 0.80*#	3.64 ± 0.82*#	0.28
Δ baseline– 6 months	1.52 ± 0.77#	1.38 ± 0.60#	0.26
REC (mm)			
Baseline	0.71 ± 0.62	0.86 ± 0.82	0.26
3 months	0.77 ± 0.54	1.07 ± 0.74*	0.09
Δ baseline–3 months	0.07 ± 0.30	0.21 ± 0.32	0.08
6 months	0.84 ± 0.66	1.12 ± 0.73*	0.11
Δ baseline–6 months	0.13 ± 0.42	0.27 ± 0.39	0.15
CAL (mm)			
Baseline	5.71 ± 0.79	5.87 ± 1.02	0.29
3 months	4.39 ± 1.09*	5.04 ± 1.00*	0.03
Δ baseline–3 months	1.32 ± 0.72	0.83 ± 0.49	0.01
6 months	4.25 ± 1.29*	4.77 ± 1.02*#	0.09
Δ baseline–6 months	1.46 ± 0.92	1.12 ± 0.54#	0.07
FMBOP (%)			
Baseline	26.1 ± 14.5	32.5 ± 17.5	0.11
3 months	13.1 ± 5.91*	19.0 ± 7.21*	0.004
Δ baseline–3 months	13.0 ± 13.6	13.5 ± 18.1	0.46
6 months	12.8 ± 6.96*	16.9 ± 6.82*	0.04
Δ baseline–6 months	13.3 ± 13.5	15.7 ± 18.8	0.33
FMPI (%)			
Baseline	32.1 ± 20.6	39.9 ± 22.6	0.13
3 months	16.5 ± 8.10*	26.8 ± 17.5*	0.01
Δ baseline–3 months	15.6 ± 18.5	13.1 ± 20.4	0.35
6 months	16.8 ± 13.9*	25.8 ± 19.2*	0.05
Δ baseline–6 months	15.4 ± 19.8	14.1 ± 22.1	0.43
Closed pockets (%) with PD ≤ 4 mm and no BOP			
Baseline	0	0	-
3 months	59.9 ± 16.2	49.8 ± 12.0	0.02
6 months	62.1 ± 18.5	56.5 ± 15.2	0.15

*Abbreviations*: *PD* probing depth, *REC* gingival recession, *CAL* clinical attachment level, *BOP* bleeding on probing, *FMPI* full-mouth plaque index

Intra-group comparison: * *p* ≤ 0.05 compared to baseline; # *p* ≤ 0.05 compared to 3 months

When compared between the groups for the secondary outcomes at 3 month, statistically significant differences were demonstrated for BOP between test and control groups (13.1% vs. 19.0%, respectively) and CAL (4.39 mm vs. 5.04 mm, respectively) together with significantly higher rates of closed pockets (PD ≤ 4 mm and no BOP) (59.9% *vs* 49.8%). Δ baseline–3 months differences of clinical parameters were all in favor of intervention group, but only CAL changes were statistically significant (*p* = 0.01), reaching 1.32 ± 0.72 mm for the test group and 0.83 ± 0.49 mm for the control group (Table 2). At 6 months, additional improvement of all periodontal parameters was demonstrated in both groups compared to the baseline (Table 2). When compared between the groups at 6 months, statistically lower scores for BOP and FMPI were demonstrated in the test group (12.8% and 16.8%, respectively) compared to 16.9% and 25.8% in the control group. Another secondary clinical variables were not statistically different between the groups at 6 months. Similarly to 3-month outcomes, Δ baseline–6 months changes were in favor of intervention group but not statistically significant.

Additionally, we analyzed mean values of clinical parameters and their Δ separately for moderate (PD 4–5 mm) and deep (PD ≥ 6 mm) pockets. For moderate pockets at 3 and 6 months, no statistically significant differences were demonstrated between the groups (Table 3).

**Table 3:** Mean scores of clinical parameters and their Δ for PD 4–5 mm at baseline and 3 and 6 months in the test (SRP plus fish oil) and control (SRP alone) groups. Data are mean ± SD

Variables	Test group *n* = 20	Control group *n* = 20	Inter-group comparisons *p* value
No PD 4 – 5 mm			
Baseline	38.2 ± 12.7	34.6 ± 12.9	0.19
3 months	17.5 ± 11.4*	17.3 ± 9.9*	0.48
Δ baseline–3 months	20.7 ± 7.57	17.3 ± 7.7	0.08
6 months	15.8 ± 11.2*#	14.4 ± 12.5*#	0.35
Δ baseline–6 months	22.4 ± 8.15#	20.2 ± 10.9#	0.24
PD (mm)			
Baseline	4.48 ± 0.13	4.42 ± 0.13	0.09
3 months	3.36 ± 0.53*	3.54 ± 0.52*	0.15
Δ baseline–3 months	1.12 ± 0.66	0.88 ± 0.52	0.09
6 months	3.23 ± 0.65*	3.30 ± 0.63*#	0.38
Δ baseline– 6 months	1.25 ± 0.68	1.13 ± 0.66#	0.29
REC (mm)			
Baseline	0.91 ± 0.58	1.05 ± 0.77	0.26
3 months	1.03 ± 0.76	1.09 ± 0.75	0.40
Δ baseline–3 months	0.12 ± 0.66	0.04 ± 0.37	0.33
6 months	0.97 ± 0.65	1.21 ± 0.75*	0.15
Δ baseline–6 months	0.06 ± 0.47	0.16 ± 0.39	0.24
CAL (mm)			
Baseline	5.39 ± 0.62	5.48 ± 0.75	0.35
3 months	4.39 ± 1.05*	4.63 ± 0.91*	0.22
Δ baseline–3 months	1.00 ± 0.92	0.85 ± 0.59	0.26
6 months	4.20 ± 1.05*	4.51 ± 0.94*	0.17
Δ baseline–6 months	1.19 ± 0.87	0.97 ± 0.68	0.19
BOP (%)			
Baseline	47.1 ± 13.7	53.1 ± 17.7	0.14
3 months	24.1 ± 13.5*	27.0 ± 9.8*	0.20
Δ baseline–3 months	23.4 ± 18.9	25.3 ± 20.9	0.36
6 months	24.0 ± 14.5*	27.2 ± 11.5*	0.23
Δ baseline–6 months	23.3 ± 20.0	25.2 ± 26.1	0.37
Closed pockets (%) with PD ≤ 4 mm and no BOP			
Baseline	0	0	-
3 months	63.0 ± 19.5	60.4 ± 10.3	0.30
6 months	63.2 ± 18.7	65.0 ± 11.6	0.36

*Abbreviations*: PD probing depth, *REC* gingival recession, *CAL* clinical attachment level, *BOP* bleeding on probing

Intra-group comparison: * *p* ≤ 0.05 compared to baseline; # *p* ≤ 0.05 compared to 3 months

However, when compared changes in the initially deep pockets, differences between the groups were evident (Table 4). At 3 months in the test group, PD reduced from 6.42 ± 0.31 mm to 4.35 ± 0.98 mm, while in the controls from 6.62 ± 0.39 mm to 5.04 ± 0.71 mm, and the difference was statistically significant (*p* = 0.01). Δ baseline–3 months PD difference was also significantly (*p* = 0.04) in favor of intervention group (2.07 ± 0.96 mm vs. 1.58 ± 0.71 mm). Thus, omega-3 PUFAs supplementation resulted in additional PD improvement of 0.49 mm. At 6 months, additional significant improvement of PD compared to the 3 months was demonstrated only in the control group, and the mean PD values were not significantly different (*p* = 0.16) between the test and control groups (4.17 ± 0.96 mm vs. 4.47 ± 0.86 mm). Accordingly, the mean change (Δ) of PD in the test group compared to the control group was not significantly different at 6 months.

**Table 4:** Mean scores of clinical parameters and their Δ for PD ≥ 6 mm at baseline and 3 and 6 months in the test (SRP plus fish oil) and control (SRP alone) groups. Data are mean ± SD

Variables	Test group *n* = 20	Control group *n* = 20	Inter-group comparisons *p* value
No PD ≥ 6 mm			
Baseline	17.3 ± 17.1	15.6 ± 14.2	0.37
3 months	12.2 ± 11.3*	12.6 ± 11.5*	0.46
Δ baseline–3 months	5.05.7 ± 11.0	3.00 ± 3.5	0.22
6 months	11.2 ± 10.9*#	11.4 ± 11.2*#	0.48
Δ baseline–6 months	6.05 ± 12.7#	3.17 ± 2.5#	0.18
PD (mm)			
Baseline	6.42 ± 0.31	6.62 ± 0.39	0.05
3 months	4.35 ± 0.98*	5.04 ± 0.71*	0.01
Δ baseline–3 months	2.07 ± 0.96	1.58 ± 0.71	0.04
6 months	4.17 ± 0.96*	4.47 ± 0.86*#	0.16
Δ baseline– 6 months	2.25 ± 0.86	2.15 ± 0.78#	0.36
REC (mm)			
Baseline	0.83 ± 0.81	1.11 ± 1.06	0.18
3 months	0.89 ± 0.94	1.50 ± 0.91*	0.03
Δ baseline–3 months	0.15 ± 0.55	0.39 ± 0.84	0.16
6 months	0.96 ± 1.18	1.59 ± 0.78*	0.03
Δ baseline–6 months	0.22 ± 0.73	0.48 ± 0.85	0.17
CAL (mm)			
Baseline	7.25 ± 0.93	7.74 ± 1.09	0.08
3 months	5.37 ± 1.30*	6.54 ± 1.17*	0.003
Δ baseline–3 months	1.88 ± 0.96	1.19 ± 1.01	0.02
6 months	5.26 ± 1.42*	6.06 ± 0.91*#	0.02
Δ baseline–6 months	1.99 ± 0.88	1.68 ± 0.94#	0.15
BOP (%)			
Baseline	58.4 ± 26.6	63.2 ± 22.5	0.28
3 months	35.0 ± 17.6*	44.1 ± 16.9*	0.05
Δ baseline–3 months	23.4 ± 25.5	19.1 ± 32.2	0.33
6 months	26.2 ± 19.9*	35.3 ± 18.1*	0.06
Δ baseline–6 months	32.3 ± 30.2	27.4 ± 28.3	0.31
Closed pockets (%) with PD ≤ 4 mm and no BOP			
Baseline	0	0	-
3 months	37.5 ± 25.5	26.4 ± 12.4	0.05
6 months	50.2 ± 24.1#	38.3 ± 22.6#	0.05

*Abbreviations*: *PD* probing depth, *REC* gingival recession, *CAL* clinical attachment level, *BOP* bleeding on probing

Intra-group comparison: * *p* ≤ 0.05 compared to baseline; # *p* ≤ 0.05 compared to 3 months

For the secondary outcome differences, mean values of REC, CAL and BOP were significantly better in the test group in comparison to the control group at 3 months, as well as the Δ baseline–3 months for CAL. Moreover, 37.5% of deep pockets were closed (PD ≤ 4 mm and no BOP) in the test group after 3 months, while in the control group only 26.4%; the difference was statistically significant (*p* = 0.05). Additional improvement of CAL in the test group was 0.69 mm. At 6 months, mean values of REC and CAL were significantly better in the test group in comparison to the control group, while the Δ baseline–6 months differences were in favor of intervention group, but not statistically significant (Table 4). Moreover, 50.2% of deep pockets were closed in the test group, while in the control group only 38.3%; the difference was statistically significant (*p* = 0.05).

Finally, we analyzed how treatment endpoint (PD ≤ 4 mm without BOP) was achieved dependent on the patients’ diagnosis and other factors affecting treatment outcome. In the test group, significantly more closed pockets demonstrated patients with stage III grade B/C periodontitis and sites associated with single-rooted teeth in comparison to the control group (Table 5). Moreover, an intra-group comparison revealed that retreatment of active pockets at 3 months resulted in significant improvement of the percentage of closed pockets in the patients with stage IV periodontitis and in the single-rooted teeth in both groups. The lowest rates of closed pockets were found at the sites associated with furcation grade III, vertical bone loss ≥ 3 mm and stage IV grade B diagnosis in both groups.

**Table 5:** Closed pockets (%) with PD ≤ 4 mm and no BOP at 3 and 6 months in the test (SRP plus FO) and control (SRP alone) groups. Data are mean ± SD

**Variables**	**Test group ***n* = 20		**Control group ***n* = 20		**Inter-group comparisons *p* value**	
	**3 months**	**6 months**	**3 months**	**6 months**	**3 months**	**6 months**
**Stage III grade B**	62.6 ± 18.22	66.7 ± 19.20	41.7 ± 14.37	43.6 ± 15.25	0.0003	0.0001
**Stage III grade C**	60.7 ± 16.78	61.4 ± 17.04	40.1 ± 11.29	46.4 ± 16.17	0.0001	0.0032
**Stage IV grade B**	39.0 ± 12.24	51.4 ± 15.38^#^	30.0 ± 11.01	45.0 ± 13.88^#^	0.0886	0.1956
**Stage IV grade C**	53.5 ± 18.37	61.2 ± 17.71^#^	55.2 ± 15.41	68.8 ± 19.77^#^	0.3768	0.0966
**Single-rooted**	56.6 ± 16.24	70.9 ± 19.10^#^	41.2 ± 10.89	51.1 ± 16.76^#^	0.0001	< 0.00001
**Multi-rooted**	53.5 ± 19.51	57.9 ± 15.63	50.2 ± 12.21	55.2 ± 15.29	0.1827	0.2469
**F1**	52.0 ± 13.45	56.0 ± 16.42	45.2 ± 13.18	61.3 ± 18.04	0.3022	0.3535
**F2**	61.0 ± 17.01	58.5 ± 18.54	48.9 ± 16.24	51.1 ± 16.43	0.1313	0.2563
**F3**	14.3 ± 6.88	28.6 ± 9.23	16.7 ± 5.69	16.7 ± 6.94	0.4420	0.31040
**VBL ≥ 3 mm**	41.7 ± 17.42	47.2 ± 16.13	34.8 ± 10.99	52.2 ± 14.15	0.2967	0.35460

*Abbreviations*: *PD* probing depth, BOP bleeding on probing, *F* furcation, *VBL* vertical bone loss

^#^*p* < 0.05 compared to 3 months for intra-group comparison

Taken together, we found that patients receiving omega-3 PUFAs demonstrated better improvement in resolution of inflammation (greater reduction of BOP), and better PD reduction and CAL gain in the initially deep pockets (PD ≥ 6 mm). However, when all active pockets were taken into account, differences of other clinical parameters between the groups were shown predominantly very short term (after 3 months), while after 6 months, there were hardly any clinical differences between the groups. Additionally, better treatment outcome was found in the patients supplemented with FO with stage III grade B/C periodontitis and at the sites associated with single-rooted teeth.

### Omega-3 PUFAs facilitate key periodontal pathogen clearance

We also examined the effect of omega-3 PUFAs on the efficacy of periopathogen clearance after non-surgical therapy. The numbers of the following bacteria were analyzed: *Phorphyromonas gingivalis, Tanarella forsythia, Treponema denticola and Aggregatibacter actinomycetemcomitans*.

**Fig. 2:**
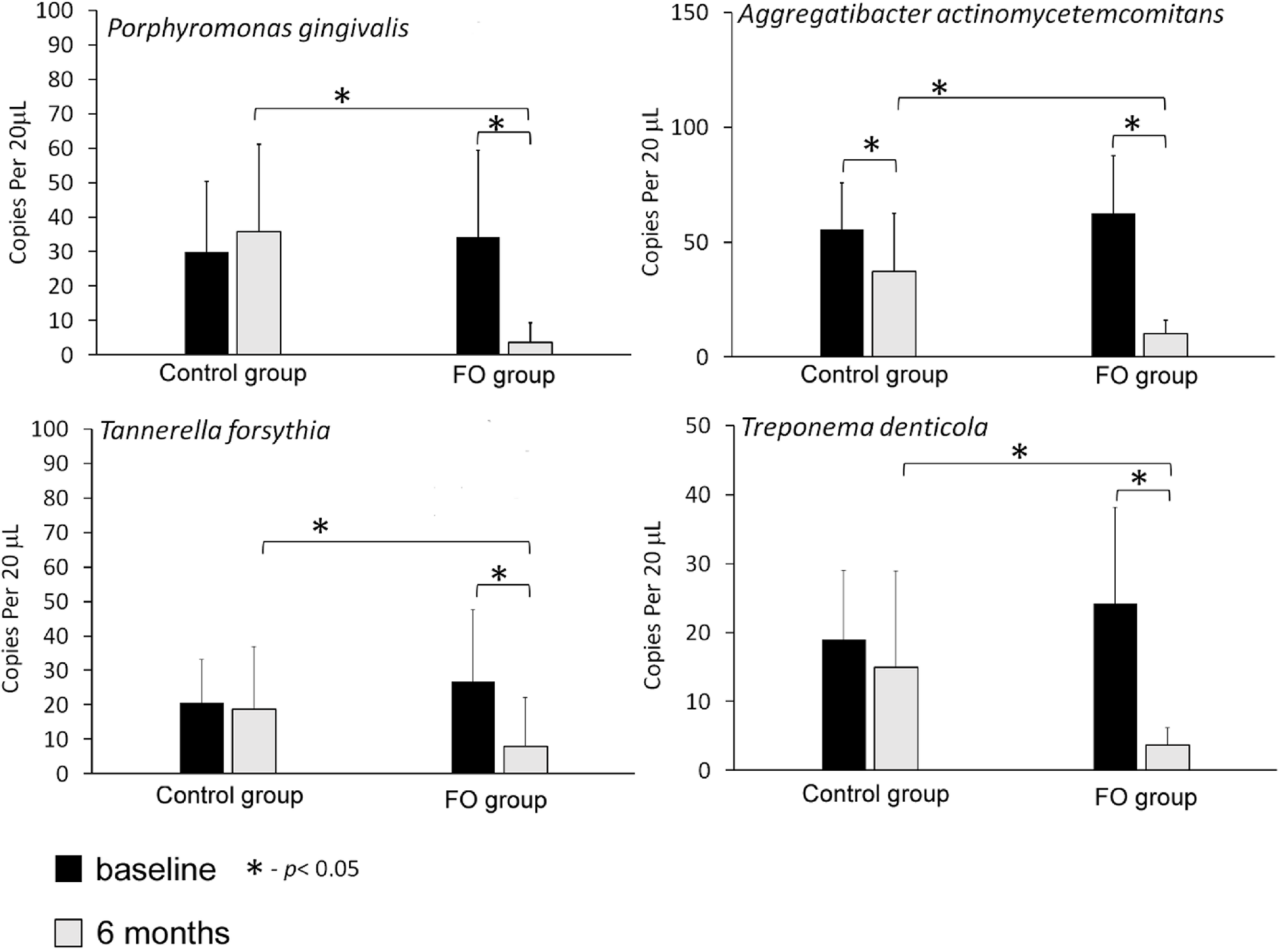
The effect of FO intake on subgingival periodontal pathogen counts. Data are mean ± SD

At baseline, no statistically significant differences in the numbers of bacteria were demonstrated between the groups (Fig. 2). At 6 months, the number of all periopathogens decreased significantly in the test group in comparison to the baseline, while in the control group only the number of *A. actinomycetemcomitans* dropped significantly. When compared between the groups at 6 months, the mean numbers of bacteria were significantly lower in the test group in comparison to the control group.

### FO intake results in increased proportions of serum n-3 PUFAs and decreased proportions of n-6 PUFAs

To define the changes in the serum lipids in the test group after 6-month supplementation with FO in relation to the baseline and to the control group, gas chromatography/mass spectrometry analysis was performed. Baseline serum lipid composition did not differ between the groups (Table 6). In the test group at 6 months, a significant increase in serum pentadecanoic acid (C15:0), stearic acid (C18:0), 5,8,11,14,17-eicosapentaenoic acid (C20:5n3) and 4,7,10,13,16,19-docosahexaenoic acid (C22:6n3) was noted, while serum levels of palmitic acid (C16:0), palmitoleic acid (C16:1n7), 10-heptadeconois acid (C17:1n7), oleic acid (C18:1n9c), 7-octadecenoic acid (C18:1n11), linolelaidic acid (C18:2n6t), linoleic acid (C18:2n6t), γ-linolenic acid (C18:3n6) and 8,11,14-eicosatrienoic acid (C20:3n6) were significantly decreased in comparison to the baseline (Table 6). The main differences corresponded with the FO composition. In the control group at 6 months, only two compounds were significantly different in comparison to the baseline: the proportions of heptadeconoic acid (C17:0) were increased, and the proportions of pentadecanoic acid (C15:0) were decreased (Table 6).

**Table 6:** The effect of FO intake on the serum lipid composition. Data are presented as the percentage of serum lipids and their Δ

	Lipid acid	Test group (*n* = 20)			Control group (*n* = 20)			FO composition
		baseline Mean (SD)	6 months Mean (SD)	Δ	baseline Mean (SD)	6 months Mean (SD)	Δ	
**1**	Myristic acid (C14:0)	0.66 (0.50)	0.63 (0.37)	-0.03	0.82 (0.46)	0.78 (0.56)	-0.06	1.63
2	Myristoleic acid (C14:1 n5)	0.05 (0.009)	0.05 (0.008)	0.004	0.03 (0.005)	0.04 (0.003)	0.02	n.d
3	Pentadecanoic acid (C15:0)	0.29 (0.59)	3.851 † # (2.96)	3.56 †	0.31 (0.17)	0.14 # (0.12)	-0.16	0.32
4	Palmitic acid (C16:0)	32.97 (6.84)	28.66 † # (10.75)	-4.31 †	32.60 (6.24)	33.34 (5.62)	0.74	11.30
5	Palmitoelaidic acid (C16:1 n7t)	0.19 (0.07)	0.16 (0.02)	-0.04	0.12 (0.07)	0.13 (0.05)	0.008	n.d
6	Palmitoleic acid (C16:1 n7)	1.20 (0.35)	0.81 # (0.03)	-0.39	1.59 (0.85)	1.52 (1.04)	-0.07	2.988
7	Heptadecanoic acid (C17:0)	0.15 (0.15)	0.13 (0.07)	-0.02	0.14 (0.10)	0.35 # (0.79)	0.21	n.d
8	Heptadec-10-enoic acid (C17:1 n7)	0.25 (0.19)	0.06 # (0.001)	-0.20	0.08 (0.05)	0.06 (0.03)	-0.03	n.d
9	Stearic acid (C18:0)	23.91 (10.22)	32.53 † # (14.49)	8.62 †	23.64 (9.30)	20.34 (10.72)	-3.30	3.95
10	Elaidic acid (C18:1n9t)	0.40 (0.10)	0.37 (0.15)	-0.02	0.42 (0.18)	0.44 (0.22)	0.03	n.d
11	Petroselinic acid (C18:1n6)	n.d	n.d		n.d	n.d		0.36
12	Oleic acid (C18:1n9c)	21.90 (8.0)	16.29 † # (5.22)	-5.61 †	21.40 (7.27)	22.40 (4.85)	1.0	17.61
13	Octadec-7-enoic acid (C18:1 n11)	0.22 (0.11)	0.07 † # (0.02)	-0.15	0.24 (0.12)	0.24 (0.12)	0.005	5.42
14	Linolelaidic acid (C18:2n6t)	0.26 (0.11)	0.15 † # (0.07)	-0.11	0.37 (0.23)	0.45 (0.12)	0.08	n.d
15	Linoleic acid (C18:2n6)	15.58(4.59)	12.12 † # (3.29)	-3.46 †	15.46 (6.79)	15.50 (7.06)	0.003	2.08
16	Arachidic acid (C20:0)	0.15 (0.09)	0.18 (0.07)	0.03	0.55 (0.48)	0.18 (0.12)	-0.37	0.61
17	gamma-Linolenic acid (C18:3n6)	0.12 (0.11)	0.06 † # (0.04)	-0.07 †	0.12 (0.08)	0.12 (0.08)	0.005	n.d
18	11-Eicosenoic acid (C20:1n9)	0.15 (0.04)	0.14 (0.05)	-0.01	0.18 (0.05)	0.12 (0.08)	-0.06	6.86
19	alfa-Linolenic acid (C18:3n3)	0.11 (0.06)	0.12 † (0.07)	0.006	0.23 (0.18)	0.23 (0.13)	0.005	0.78
20	Rumenic acid (C18:2n7c9t11)	0.21 (0.10)	0.14 (0.05)	-0.07	0.25 (0.10)	0.19 (0.11)	-0.06	n.d
21	Octadeca-10,​12-​dienoic acid (C18:2 n6 t10c12)	0.22 (0.18)	0.20 (0.08)	-0.03	0.24 (0.12)	0.27 (0.13)	0.03	n.d
22	Eicosa-11,14-dienoic acid (C20:2 n6)	0.05 (0.02)	0.07 (0.07)	0.02	0.1 (0.07)	0.1 (0.09)	0.01	n.d
23	Behenic acid (C22:0)	0.06 (0.04)	0.06 (0.04)	0.001	0.07 (0.09)	0.07 (0.05)	-0.001	n.d
24	Eicosa-13,16-dienoic acid (C20:2 n4)	0.10 (0.06)	n.d	0	0.08 (0.04)	0.10 (0.07)	0.02	n.d
25	Eicosa-8,11,14-trienoic acid (C20:3 n6)	0.45 (0.17)	0.16 † # (0.08)	-0.29 †	0.44 (0.13)	0.36 (0.13)	-0.07	n.d
26	Tricosanoic acid (C23:0)	n.d	n.d		0.04 (0.02)	0.08 (0.04)	0.04	n.d
27	Arachidonic acid (C20:4n6)	1.57 (0.68)	1.53 † (0.65)	-0.03 †	1.34 (0.74)	2.25 # (0.75)	0.91	1.13
28	Stearidonic acid (C18:4 n3)	n.d	n.d		0.04 (0.02)	0.01 (0.005)	-0.03	0.85
29	Lignoceric acid (C24:0)	0.04 (0.03)	0.09 † (0.03)	0.05 †	0.09 (0.03)	0.03 (0.007)	-0.06	n.d
30	Eicosa-5,8,11,14,17-pentaenoic acid (C20:5n3)	0.22 (0.04)	0.80 † # (0.55)	0.62 †	0.25 (0.06)	0.32 (0.22)	0.07	17.08
31	Cetoleic acid (C22:1n 11)	n.d	n.d		n.d	n.d		3.39
32	Docosapentaenoic acid	n.d	n.d		n.d	n.d		1.06
33	Docosa-4,7,10,13,16,19-hexaenoic acid (C22:6n3)	0.10 (0.12)	0.38 † # (0.30)	0.28 †	0.13 (0.10)	0.21 (0.19)	0.07	9.03
34	Erucic acid (C22:1n9)	n.d	n.d		n.d	n.d		0.80
35	Squalene	n.d	n.d		n.d	n.d		8.63
36	Nervonic acid (C24:1n9)	0.13 (0.04)	0.06 (0.01)	-0.06	0.12 (0.005)	0.11 (0.04)	0.006	0.47

*n.d* non-detectable (< 0.03%)

^†^*p* ≤ 0.05 compared to the control group

^#^*p* ≤ 0.05 compared to baseline

The inter-group comparison at 6 months demonstrated significantly higher proportions of pentadecanoic acid (C15:0), stearic acid (C18:0), 5,8,11,14,17-eicosapentaenoic acid (C20:5n3) and 4,7,10,13,16,19-docosahexaenoic acid (C22:6n3) in the test group in comparison to the control group. On the contrary, the proportions of n-6 PUFAs palmitic acid (C16:0), oleic acid (C18:1n9c), linolelacidic acid (C18:2n6t), linoleic acid (C18:2n6t), γ-linolenic acid (C18:3n6), arachidonic acid (C20:4n6) and 8,11,14-eicosatrienoic acid (C20:3n6) were significantly lower in the test group compared to the control group at 6 months.

## Discussion

In this study, we demonstrated that SRP with adjunct supplementation with fish oil rich in omega-3 PUFAs for 6 months had potential to induce measurable clinical and microbiological outcomes in comparison to SRP alone. In our study, patients were supplemented with higher doses of omega-3 PUFAs (2.6 g of EPA and 1.8 g of DHA) compared to previous studies [24–29].

We revealed beneficial changes in all clinical parameters in both groups at 3 months (after the initial therapy) and at 6 months (after residual pocket therapy) compared to that at baseline. The differences between the groups in favor of the test group were statistically significant mainly at 3 months and in the initially deep pockets (PD ≥ 6 mm). In our previous study assessing an effect of omega-3 PUFAs following the initial periodontal therapy [36], a statistically significant reduction in BOP, improvement of CAL and higher percentage of closed pockets with probing depth ≤ 4 mm and no BOP were found in the test group vs. control group 3 months following treatment, which is very comparable to the present findings. Our results after 3 months are in accordance with the recent systematic review and meta-analysis [37] assessing the use of omega-3 PUFAs adjunctively to non-surgical treatment of periodontitis. Authors revealed significant overall PD reduction of 0.42 mm and CAL gain of 0.58 mm, and concluded that the use of omega-3 fatty acids during non-surgical treatment of periodontitis can provide additional benefits in CAL gain and PD reduction compared with subgingival instrumentation alone.

At 6 months, only BOP scores were statistically different when all pockets in the test and control groups were considered. This indicates that supplementation with high-dose omega-3 PUFA, has detectable but limited clinical effect in the treatment of generalized periodontitis stage III and IV. A deteriorating compliance to the treatment regimen during 6-month period may be a probable explanation that clinical parameters were not significantly better at 6 months in the test group in comparison to the control group. It is well-known that adherence to medical treatment is one of the issues in the treatment of chronic diseases. It was demonstrated that nearly 50% of patients failed to adhere to their medical directives, and that 25% of patients were non-adherent to prevention and disease management activities including medication taking, appointment keeping, screening, exercise, and dietary changes [38, 39].

According to 2017 World Workshop of EFP, BOP is an important clinical parameter related to assessment of periodontitis treatment outcomes and post-treatment residual disease risk [35]. Therefore, lower BOP rates observed at the end of our study and higher rates of closed pockets at 3 and 6 months in initially deep pockets, in the patients with stage III periodontitis and at the sites associated with single-rooted teeth in the test group were desirable treatment outcomes that could provide long-term disease resolution and less need for retreatment. Reduced BOP rates in the test group might be attributed to the anti-inflammatory properties of omega-3 PUFAs. We have previously demonstrated that the levels of proinflammatory IL-8 and IL-17 were markedly lower, and the level of anti-inflammatory IL-10 was significantly higher in the salivary samples of the patients that received omega-3 PUFAs for 3 months in comparison with the patients treated with SRP alone [36].

Our results obtained at 3 months are in accordance with the study of Deore et al., demonstrating that supplementation with omega-3 PUFA during non-surgical periodontal treatment significantly reduced GI, BOP, PD and CAL [25]. However, our cohorts demonstrated a more severe form of periodontitis, as the initial mean PD in the test and control groups was 5.01 ± 0.49 and 5.02 ± 0.71, while in the study of Deore et al., 4.26 ± 1.10 and 4.05 ± 1.03, respectively. At 6 months, others demonstrated similar clinical results as ours. Keskiner et al. showed no statistically significant differences in clinical parameters like PD, CAL, BOP and GI between the test and control groups [24]. However, patients received anecdotally low dose of omega-3 PUFAs (19.19 mg of DHA and 6.25 mg of EPA twice daily). Similarly, Martinez et al. revealed no significant differences in the clinical parameters between the test and placebo group at 4 and 12 months independently on the initial PD, but again, patients received rather low doses of omega-3 PUFAs (180 mg of EPA and 120 mg of DHA three times a day) [26].

In our study, we also analyzed mean scores of clinical parameters separately for moderate (4–5 mm) and deep (≥ 6 mm) pockets. Inter-group comparison for deep pockets after 3 months showed statistically significant differences between groups for PD, REC and their Δ, and percentage of closed pockets, in favor of the test group. Moreover, assessment after 6 months revealed statistically significant differences between the groups for REC and CAL, and in the number of closed pockets also in favor of the test group. However, inter-group comparison for their Δ baseline–6 months did not reveal statistical differences. When comparing our clinical results with the recent systematic review and meta-analysis on subgingival instrumentation in the treatment of periodontitis [40], mean PD reduction at 3 and 6 months was better in the FO group, 1.3 mm and 1.5 mm, respectively, in comparison to 1.0 mm and 1.4 mm shown in the meta-analysis at 3–4 or 6–8 months, respectively. Furthermore, considering the extent of disease resolution, as measured in terms of percentage of closed pockets, in the FO group 60% of pockets were closed at 3 months and 62% at 6 months in comparison to 57% at 3–4 months and 74% at 6–8 months shown in the meta-analysis [40].

In this study, we also addressed an antimicrobial effect of omega-3 PUFAs. Quantitative analysis of crucial periodontal pathogenic bacteria *P. gingivalis*, *T. forsythia*, *T. denticola* and *A. actinomycetemcomitans* demonstrated significantly lower numbers of these bacteria in the FO group in comparison to the control group at 6 months. Surprisingly, an intra-group analysis failed to demonstrate statistically significant differences in bacterial counts in the control group, except for *A. actinomycetemcomitans.* However, some studies applying RT-qPCR already demonstrated no reduction or even an increase in red complex bacteria following non-surgical therapy [41–43]. It was suggested that missing correlation between the microbiological and clinical parameters could be explained by the fact that periodontal treatment cannot eradicate, but only reduce, periopathogens. An alternative explanation could be sampling fluctuation at the infection/probing sites [43], especially providing that we demonstrated higher FMPI in the control group at all time points.

While omega-3 PUFA anti-inflammatory activity is well-described, little is known about their effect on the growth and survival of oral bacteria. Recently, it was demonstrated that omega-3 PUFAs and their ester derivatives exhibited strong in vitro antimicrobial activity against various oral pathogens, such as *Candida albicans* and periodontopathogens, *A. actinomycetemcomitans*, *F. nucelatum*, and *P. gingivalis* [44, 45]. Moreover, Ribeiro-Vidal et al. provided evidence that application of EPA or DHA extracts on in vitro multispecies biofilm model of six bacterial species (*S. oralis*, *A. naeslundii*, *V. parvula*, *F. nucleatum*, *P. gingivalis* and *A. actinomycetemcomitans*) for 60 s resulted in an antimicrobial effect [46]. Contrary to those in vitro studies, in a 3-month pilot randomized controlled trial, 2 g of daily DHA with 81 mg of aspirin did not significantly modify total bacterial plaque growth in the patients that were not subjected to mechanical plaque removal [47]. However, an inhibition of *P. gingivalis* growth was detected, whereas in the placebo group, an increase in the mean counts of *P. gingivalis* was observed. Furthermore, among all investigated bacterial complexes, red complex tended to drop to a higher degree than the others [47]. Potential antimicrobial action of omega-3 PUFAs against periodontal pathogens seems to be crucial not only in the context of treatment of periodontitis. *P. gingivalis*, *T. denticola* and *A. actinomycetemcomitans* were shown to be linked to various systemic diseases, including cardiovascular diseases, Alzheimer’s disease, rheumatoid arthritis and systemic lupus erythematosus [48, 49]. Thus, decrease of key periodontal bacteria abundance support periodontal treatment and protect from other chronic diseases.

Animal studies provided more evidence for possible role of omega-3 PUFAs in bacterial clearance in periodontitis. It was demonstrated that topical treatment with RvE1 inhibited tissue and bone damage, and resulted in eradication of *P. gingivalis* [50]. Authors hypothesized that resolution of inflammation effectively eliminates *P. gingivalis* from the lesion by removing the nutritional source for the bacteria. In another study with experimental periodontitis, marked changes in subgingival microbiota after RvE1 treatment were revealed [51]. Authors also suggested that resolution of local inflammation had a major role in shaping the composition of the subgingival microbiota. In our study at 6 months, the level of key periopathogens was reduced significantly only in the test group, but microbiological result was not in accordance with clinical improvement in the control group, thus inhibition of bacterial growth cannot be attributed to the resolution of inflammation only. Although bacterial clearance was combined with statistically significant CAL improvement in deep pockets ≥ 6 mm at 3 and 6 months and with statistically significant PD reduction at 3 months in the FO group, overall clinical improvement in the control group should be accompanied by better reduction of subgingival periopathogens. Therefore, it seems that in our study, omega-3 PUFAs had an activity against periodontal bacteria independently of their anti-inflammatory effects promoting resolution of inflammation, and, in a consequence, inhibition of ‘inflammophilic’ bacteria.

Optimal plaque control is crucial factor of long-term effects of periodontal treatment. In this study, we demonstrated a statistical difference in FMPI between groups at 3 and 6 months. This difference may result from better motivation and involvement in the treatment process of the patients from intervention group, because they received fish oil as an adjunct to SRP. Alternatively, fish oil intake may interfere with supragingival dental plaque formation resulting in lower FMPI in the test group. Nevertheless, we cannot fully exclude that better clinical and microbiological outcome in the test group may be partially attributed to lower FMPI in comparison to the control group.

In this study, we used fish-derived oil mixture as a source of EPA and DHA. Natural fish oil was chosen to mimic dietary conditions where a range of essential oils are consumed. To address, how fish oil consumption affected serum lipid composition, gas chromatography/mass spectrometry analysis was performed. We found that FO intake resulted in increased proportions of serum n-3 PUFAs and decreased proportions of n-6 PUFAs. This shift leads to increase in the proportion of EPA and DHA in the membranes of inflammatory cells, and thus by competing reduces the level of arachidonic acid [52]. This, in turn, results in decreased synthesis of pro-inflammatory mediators derived from arachidonic acid which is a major product of omega-6 PUFA metabolism in the body [53, 54].

The present study has several limitations. The first one is the initial difference between the test and control groups in terms of age. This was a result of a relatively high dropout rate, mainly due to SARS-CoV-2 pandemic-related lockdown. When the baseline data of all fifty initially enrolled participants were taken into account, no statistical differences between the groups were noted. Even though at baseline the advancement of periodontitis was similar in both groups, the treatment response might be different as periodontal tissue healing ability may deteriorate with age [55]. Another limitation is the use of fish-derived oil mixture instead of isolated EPA and DHA, thus, other components of the fish oil may affect the results of study. Specifically, squalene and alkylglycerols present in the fish oil were demonstrated to activate Th1-type IL-12 and IFN-γ cytokine production and increase antioxidant activity of serum [56, 57]. It is also worth mentioning that the amounts of vitamin A and D consumed with fish oil in the test group were 3–20 times below recommended daily doses. Since fish oil was chosen as a source of EPA and DHA in our study, no placebo was employed and patients were aware of treatment allocation. From our experience, even when fish oil is taken in capsules, its taste cannot be masked because of characteristic fish burps.

## Conclusions

In conclusion and within the limitations of this study, the adjunct use of omega-3 PUFAs during non-surgical treatment of periodontitis provided short-term clinical benefits (mainly at 3 months), especially in the deeper pockets (≥ 6 mm), in the patients with stage III grade B/C periodontitis and at the sites associated with single-rooted teeth, as well as more effective periopathogen clearance in comparison to SRP alone. Administration of fish oil containing omega-3 PUFAs for 6 months was safe and well-tolerated by the patients.

## Data Availability

The data that support the findings of this study are available on request from the corresponding author. The data are not publicly available due to privacy or ethical restrictions.
